# Synthesis, Physicochemical Characterization, Antimicrobial Properties, and DFT/ADMET Calculations of Imidazolium-Based Ionic Liquids with a Homologous Series of Oxychlorine Anions

**DOI:** 10.3390/molecules30224346

**Published:** 2025-11-10

**Authors:** Milan B. Vraneš, Eleonora Čapelja, Maja Karaman, Teona Teodora Borović, Andrija Vukov, Sara Klimenta, Vesna Rastija, Jovana J. Selak

**Affiliations:** 1 Department of Chemistry, Biochemistry and Environmental Protection, Faculty of Science, University of Novi Sad, 21000 Novi Sad, Serbia; teona@dh.uns.ac.rs (T.T.B.); andrija.vukov@dh.uns.ac.rs (A.V.); saraklimenta@hotmail.com (S.K.); jovanap@dh.uns.ac.rs (J.J.S.); 2 Department of Biology and Ecology, Faculty of Sciences, University of Novi Sad, 21000 Novi Sad, Serbia; eleonora.capelja@dbe.uns.ac.rs (E.Č.); maja.karaman@dbe.uns.ac.rs (M.K.); 3 Department of Agroecology and Environmental Protection, Faculty of Agrobiotechnical Sciences Osijek, Josip Juraj Strossmayer University of Osijek, HR-31000 Osijek, Croatia; vrastija@fazos.hr

**Keywords:** ionic liquids, transport properties, antimicrobial activity, DFT calculations, ADMET modeling

## Abstract

Imidazolium-based ionic liquids bearing a homologous series of oxychlorine anions—1-butyl-3-methylimidazolium chlorite, chlorate, and perchlorate—were synthesized and characterized to relate anion oxygenation to density, thermal expansivity, viscosity, electrical and molar conductivity, ionicity, and antimicrobial performance. Temperature-dependent measurements were carried out from 293.15 to 323.15 K: density and viscosity were recorded and modeled to obtain thermal expansion coefficients; electrical and molar conductivities were measured under identical conditions; and activation parameters were extracted by Arrhenius analysis for viscous flow and for conductivity. Ionicity was assessed from Walden plots and quantified by vertical deviation from the potassium-chloride reference (Angell approach). Complementary DFT calculations provided optimized ion-pair geometries, noncovalent contact patterns, molecular electrostatic potential maps, and frontier-orbital descriptors. In silico ADMET properties were computed to contextualize pharmacokinetic and safety flags. Antimicrobial activity was evaluated by broth microdilution against *Escherichia coli*, *Staphylococcus aureus*, *Bacillus cereus*, and *Candida quilliermondii*; [Bmim]Cl was included as a comparator to isolate the effect of anion oxygenation. The combined experimental–computational workflow delineates how chlorite, chlorate, and perchlorate shape physicochemical behavior, ionicity, and bioactivity in [Bmim] ionic liquids, providing design guidance for future applications.

## 1. Introduction

Oxychlorine anions, including chlorite ClO_2_^−^ [1], chlorate ClO_3_^−^ [2,3], and perchlorate ClO_4_^−^ [4,5], have oxidizing properties that disrupt microbial structures and functions and demonstrate broad-spectrum activity against various pathogens. Their rapid antimicrobial action at low concentrations reduces the likelihood of classical resistance and supports effectiveness in water treatment, disinfection, and sterilization [6]. The chlorite anion originates from the weak chlorous acid, while the chlorate and perchlorate anions derive from the strong acids chloric and perchloric acid. The strength of an acid reflects its ability to dissociate in water and release hydrogen ions, which determines solution acidity. Differences in acid strength directly influence the properties of the conjugate anions, including stability, reactivity, basicity, and coordination behavior. Therefore, a clear understanding of these anions is essential for interpreting their chemical behavior and applications.

When combined with the 1-butyl-3-methylimidazolium cation, BMIM, in ionic liquids, oxychlorine anions can enhance antimicrobial performance and provide a versatile platform against microbial contamination. Ionic liquids are molten salts noted for low volatility, high thermal stability, and tunable solvation and interfacial properties [7,8]. BMIM-based ionic liquids enable purposeful adjustment of physicochemical behavior to support targeted antimicrobial uses by facilitating delivery, controlling interactions at biological interfaces, and allowing dose optimization. Recent studies highlight effectiveness against various microorganisms through mechanisms that include membrane disruption and interference with metabolic processes [9]. Synergistic combinations with other antimicrobial agents or functional additives further expand potential uses in pharmaceuticals, cosmetics, food preservation, and medical devices.

The objective of this study is to synthesize three BMIM-based ionic liquids that contain a homologous series of oxychlorine anions: 1-butyl-3-methylimidazolium chlorite, 1-butyl-3-methylimidazolium chlorate, and 1-butyl-3-methylimidazolium perchlorate. BMIM chlorate and, more commonly, perchlorate have been reported previously in the literature. BMIM perchlorate was investigated as an additive for heavy oil reduction [10], as an electrolyte [11], and both BMIM perchlorate and chlorate have investigated as additives for HPLC systems [12,13]. We have been unable to find any mention of previous synthesis of BMIM chlorite and the data for BMIM perchlorate and chlorate is both limited and has a notable lack of physicochemical characterization, with only BMIM perchlorate having density and viscosity data available [11]. Therefore, we aim to fill this gap in the available literature and to elucidate their structures and physicochemical properties and to evaluate their antimicrobial activity. Another objective is to determine how increasing anion oxygenation across the chlorite–chlorate–perchlorate series influences physicochemical behavior and antimicrobial performance. Experimental results are supported by computational DFT analysis and in silico ADMET calculations to place the findings within a coherent structure–property–bioactivity framework.

## 2. Results and Discussion

### 2.1. Physicochemical Characterization

Table 1 presents the results of the physicochemical characterization for the synthesized ionic liquids, [Bmim][ClO_2_], [Bmim][ClO_3_], and [Bmim][ClO_4_], including measurements of their density, viscosity, and electrical conductivity at temperatures ranging from *T* = 293.15 to 323.15 K with 5 K intervals.

**Figure 1:**
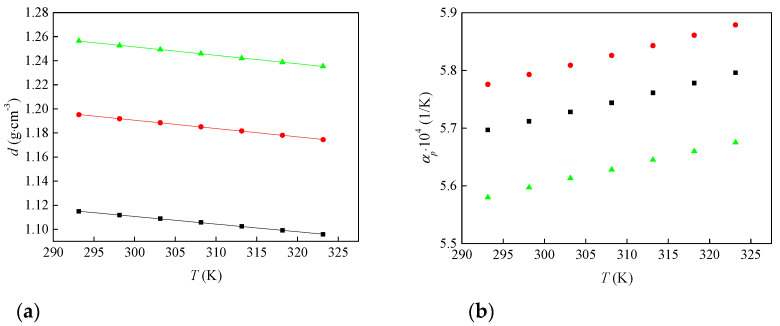
Change in (**a**) density and (**b**) thermal expansion coefficient of synthesized ionic liquids with temperature: ■—[Bmim][ClO_2_], ●—[Bmim][ClO_3_] and ▲—[Bmim][ClO_4_].

Figure 1a shows that density decreases linearly with temperature for all three ionic liquids. At any temperature, [Bmim][ClO_4_] has the highest density and [Bmim][ClO_2_] the lowest. This agrees with the higher oxygenation and symmetry of the anions. The measured densities were fitted to a linear temperature dependence according to Equation (1). The fitted parameters *a* and *b* and the regression coefficient (*R*^2^) are listed in Table 2.

*d* (g·cm^−3^) = *a* + *b* × *T* (K) (1)

Based on the temperature dependence of density, the isobaric thermal expansion coefficient *α*_p_ was calculated for all three ionic liquids according to Equation (2):

(2) $${\alpha_{p}=-\frac{1}{d}\left(\frac{\partial d}{\partial T}\right)}_{p,m}$$

**Figure 2:**
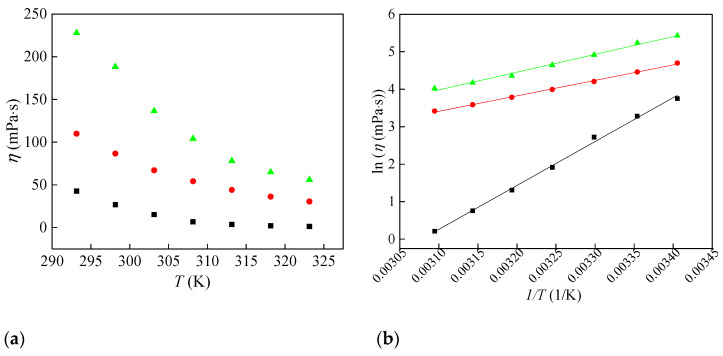
Change in (**a**) viscosity of synthesized ionic liquids with temperature and (**b**) Arrhenius plot for viscosity: ■—[Bmim][ClO_2_], ●—[Bmim][ClO_3_] and ▲—[Bmim][ClO_4_].

Figure 2b displays the temperature dependence of α_p_, and the corresponding values are listed in Table 1. The thermal expansion coefficients increase with temperature for all three ionic liquids, a common behavior attributed to weakened ion–ion interactions at higher temperatures. Within this trend, the perchlorate salt exhibits the lowest α_p_ and the slowest increase with temperature, indicating lower expansivity for [Bmim][ClO_4_] compared with [Bmim][ClO_2_] and [Bmim][ClO_3_].

The viscosity data are presented in Figure 2a. As shown in Figure 2a, viscosity decreases with increasing temperature. Further, as the oxygen content of the anion rises, the viscosity of the ionic liquids also increases. The ionic liquid [Bmim][ClO_4_] exhibits the highest viscosity values, suggesting tighter packing in the liquid structure. This trend can be attributed to the geometry and higher oxygenation of the perchlorate anion, which promotes stronger cation–anion interactions.

Viscosity values were further fitted as a function of temperature by the Arrhenius Equation (3) and the results are shown in Figure 2b.

*η* = *η*° × *e*
^*E*a/*RT*^ (3)

Taking the logarithm of the previous equation, the following equation is obtained:

ln *η* (mPa·s) = ln *η*° + *E*_a_/(*RT*) (4)

Equation (4) involved an adjustable parameter *η*°, the universal gas constant *R* (8.314 J·mol^−1^·K^−1^), and the activation energy of the viscous flow represented by *E*_a_. Table 2 provides the activation energy values for the three ionic liquids. It can be observed from the table that [Bmim][ClO_2_] has significantly higher activation energy values compared to [Bmim][ClO_3_] and [Bmim][ClO_4_]. These results indicate that viscosity in [Bmim][ClO_2_] shows the strongest temperature dependence over the examined range.

**Figure 3:**
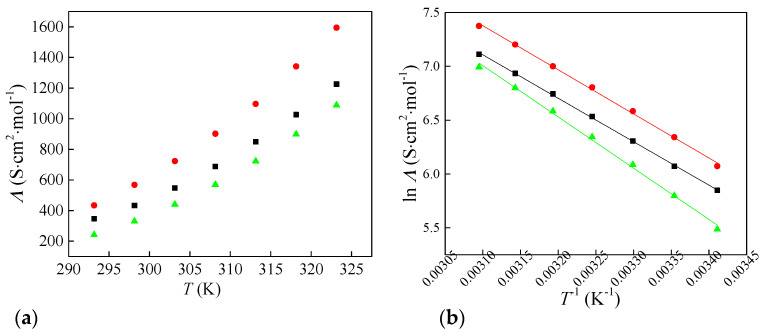
Change in (**a**) molar conductivity of synthesized ionic liquids with temperature and (**b**) Arrhenius plot for molar conductivity: ■—[Bmim][ClO_2_], ●—[Bmim][ClO_3_] and ▲—[Bmim][ClO_4_].

From the experimental values of electrical conductivity and measured density (Table 1), molar conductivities (*Λ*) were calculated using Equation (5):

(5) Λ=κM/d

where *Λ* is the molar conductivity, *k* is the measured electrical conductivity, *M* is the molar mass, and *d* is the density of the investigated ionic liquids. The calculated values of molar conductivities are shown in Figure 3a. The electrical and molar conductivity of the ionic liquid with chlorate anion is higher than that of the ionic liquid with chlorite and perchlorate anion. This maximum in *κ* and *Λ* for [Bmim][ClO_3_] plausibly reflects an optimal balance between ionicity and viscous drag across the oxychlorine series. In [Bmim][ClO_2_], stronger specific interactions with the cation and greater ion pairing reduce the number of free charge carriers despite lower viscosity. In [Bmim][ClO_4_], the weakly coordinating, highly symmetric anion favors ionicity, but the higher viscosity suppresses ion mobility. Chlorate sits between these limits: sufficient charge delocalization to limit ion pairing, yet lower viscous resistance than the perchlorate salt, resulting in the highest conductivity. This trend is consistent with the viscosity ordering and with the activation behavior of conductivity discussed below.

The molar conductivity values are also fitted as a function of temperature by the *Arrhenius* Equation (6):

*Ʌ* = *Ʌ*° × *e*^−*E’*a/*RT*^ (6)

where *Ʌ*° is the pre-exponential factor, *E*’_a_ represents the conductivity activation energy, and R is the gas constant. The conductivity activation energies for all three ionic liquids are given in Table 2. [Bmim][ClO_4_] shows the highest value, while [Bmim][ClO_2_] and [Bmim][ClO_3_] are very similar. This indicates that charge transport in the perchlorate salt is more strongly temperature-dependent and requires more thermal energy to achieve comparable ionic mobility. The result is consistent with its higher viscosity and the greater viscous hindrance to ion motion over the examined range.

**Figure 4:**
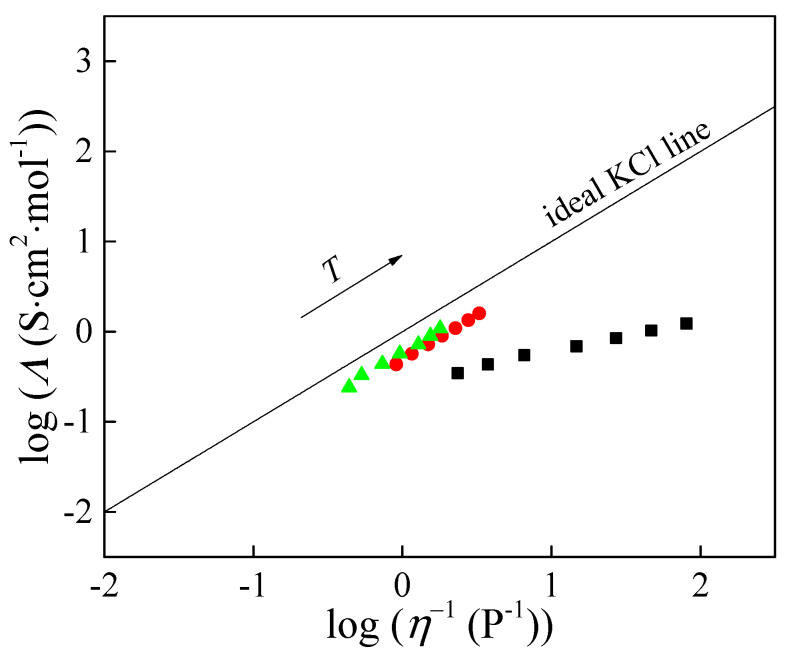
Walden diagram: ■—[Bmim][ClO_2_], ●—[Bmim][ClO_3_], and ▲—[Bmim][ClO_4_].

The Walden diagram (Figure 4) relates molar conductivity (*Λ*) to fluidity (*η*^−1^ in 1/Poise unit) [14].

The classical relation is as follows:

*Λ* × *η*^α^ = *K* = *const*. (7)

Applying the logarithm function to Equation (7), the following equation is obtained:

log *Λ* (S·cm^2^·mol^−1^) = *a* × log (*η*^−1^) + log *K* (8)

Plotting log*Λ* against the logarithm of fluidity (*η*^−1^) yields an approximately linear relationship (Figure 4). The slope of the Walden plot, *a*, expresses how strongly conductivity depends on viscosity and serves as a qualitative indicator of ion–viscosity coupling. For [Bmim][ClO_2_], the calculated value is *a* = 0.35, indicating pronounced ion association and partial decoupling of charge transport from viscous flow. By contrast, *a* ≈ 1 for [Bmim][ClO_3_] and [Bmim][ClO_4_], showing that the temperature dependence of their molar conductivity is governed almost exclusively by viscosity in the examined range.

The Walden plot also quantifies deviation from an ideal reference line defined by aqueous KCl at *c* = 0.01 mol·dm^−3^, where ions are effectively fully dissociated and changes in molar conductivity reflect viscosity alone. The deviation from the ideal KCl line (ΔW) can be calculated from the following equation [15,16,17]:

ΔW = log *η*^−1^ − log *Λ* (9)

The value of ΔW directly indicates the degree of effective dissociation and ion mobility. This deviation is a direct indicator of effective dissociation and ion mobility. Following Angell, ionicity is computed as follows:

*α* (%) = 10^−∆W^ × 100 (10)

The ionicity values obtained from Equation (10) are listed in Table 1 and show a clear separation across the series. At 323.15 K, [Bmim][ClO_3_] and [Bmim][ClO_4_] exhibit α ≈ 93% and ≈95%, respectively, remaining high and essentially stable over the examined temperature range. In contrast, [Bmim][ClO_2_] shows a much lower α, about 67% at 323.15 K, and it decreases further with temperature to about 35% at the highest temperature examined. These quantitative results are fully consistent with the Walden slopes: *a* ≈ 1 for [Bmim][ClO_3_] and [Bmim][ClO_4_] indicates that molar conductivity is governed almost entirely by viscosity, whereas *a* ≈ 0.35 for [Bmim][ClO_2_] reflects pronounced ion association and partial decoupling of charge transport from viscous flow. The conductivity activation parameters (Table 2) support the same picture, with [Bmim][ClO_2_] showing the least viscosity-controlled behavior among the three.

### 2.2. Computational Simulation Results

**Figure 5:**
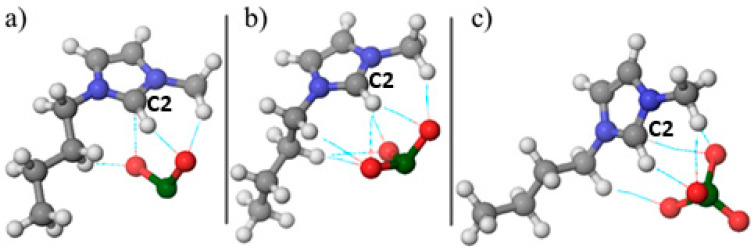
Optimized geometry of ionic liquids (**a**) [Bmim][ClO_2_], (**b**) [Bmim][ClO_3_] and (**c**) [Bmim][ClO_4_].

The initial stage of computational DFT simulations involved geometric optimization of [Bmim][ClO_2_], [Bmim][ClO_3_], and [Bmim][ClO_4_]. The optimized structures are shown in Figure 5.

The present geometric optimization results aim to investigate the alignment of chlorine oxyanion in various compounds, to understand the formation of non-covalent interactions. Figure 5 illustrates that the oxygen atoms in red orient themselves to the hydrogen atoms in white, forming these interactions, which are represented by dashed lines. Furthermore, a thermodynamic analysis of the formation of monomer units of ionic liquids was conducted, and the results are presented in Table 3.

A thermodynamic analysis was then performed to compare the separated ions with the associated ion pair. Gibbs free energy, Δ*G*, was used to evaluate the relative stability of separated ions versus the associated pair in the gas phase. In this vacuum model, [Bmim][ClO_4_] appears least specifically stabilized by ion pairing, whereas [Bmim][ClO_2_] shows the strongest specific stabilization. Positive entropy changes, Δ*S*, indicate increased disorder when ions are separate compared to the paired state. Positive enthalpy values suggest that separating the ion pair into its constituent ions is endothermic, reflecting the energy required to disrupt electrostatic and C–H···O contacts. Larger Δ*H* values point to stronger specific cation–anion stabilization in the corresponding system. These gas-phase trends should be interpreted with caution for condensed phases. To better emulate liquid-phase screening, we also report BSSE-corrected interaction energies and, where possible, calculations with a polarizable continuum.

**Figure 6:**
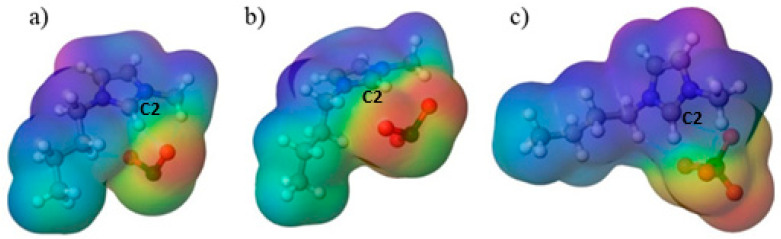
Visual representation of the molecular electrostatic potential distribution of (**a**) [Bmim][ClO_2_] (**b**) [Bmim][ClO_3_] (**c**) [Bmim][ClO_4_].

The next stage of the investigation involved calculating the molecular electrostatic potential as a descriptor of where electrostatic and hydrogen-bond interactions are most likely to occur in the investigated molecules. As shown in Figure 6, positive potential is localized on the imidazolium cation and is strongest around the imidazole ring, especially near the C2–H site, while negative potential resides on the oxygens of the oxychlorine anions. This distribution explains the directional C–H···O contacts observed in the optimized structures and is consistent with the ionicity trends: the more localized negative potential on chlorite supports stronger site-directed contacts with the cation, matching the lower ionicity and the larger deviation from the KCl ideal line, whereas the more delocalized and symmetric perchlorate shows weaker specific coordination, in line with its high ionicity and behavior closer to the ideal dependence.

In the final stage, frontier-orbital descriptors were computed. The HOMO is the highest occupied molecular orbital and the LUMO is the lowest unoccupied molecular orbital. Their energy difference defines the HOMO–LUMO gap according to Equation (11):

(11) $$\Delta E_{GAP}=E_{HOMO}-E_{LUMO}$$

**Figure 7:**
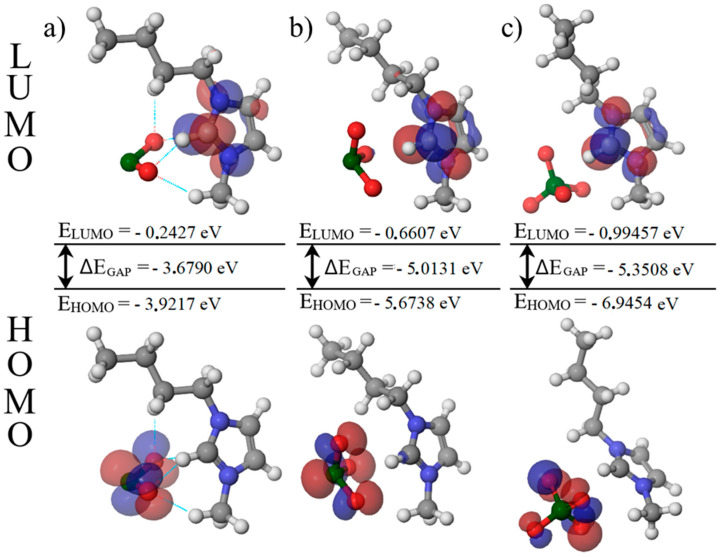
Representation of HOMO and LUMO orbitals (**a**) [Bmim][ClO_2_] (**b**) [Bmim][ClO_3_] (**c**) [Bmim][ClO_4_].

A larger gap indicates greater chemical hardness and a lower electronic response, whereas a smaller gap indicates a softer, more electronically responsive system. Figure 7 shows the largest HOMO–LUMO gap for [Bmim][ClO_4_] and the smallest for [Bmim][ClO_2_]. This electronic ordering agrees with the experimental picture: perchlorate displays high ionicity and molar conductivity mainly governed by viscosity, while chlorite shows reduced ionicity and conductivity that depend both on viscosity and on the extent of dissociation. Chlorate lies between these limits, which helps explain its highest conductivity as a balance between ionicity and viscous drag.

### 2.3. Antimicrobial Activity

**Figure 8:**
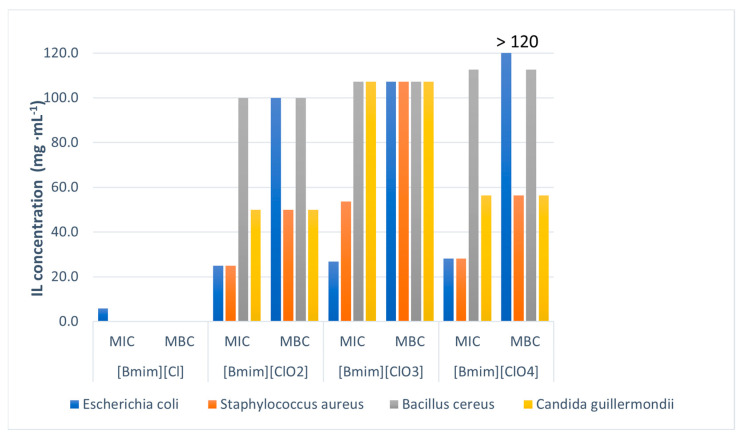
Antimicrobial activity of tested ILs against bacteria and yeast.

Results of antimicrobial testing against three bacterial species (*Escherichia coli*, *Staphylococcus aureus*, *Bacillus cereus*), and one yeast (*Candida quilliermondii*), are presented in Table 3 and Figure 8. All tested microorganisms showed sensitivity to the tested ILs within the tested concentration range. Also, it has been shown that tested ILs are more toxic if the anion is in the form of chlorite, chlorate, or perchlorate than chloride, suggesting that oxygen in the anion structure increases toxicity.

Among bacterial and yeast cells, [Bmim][ClO_2_] showed the best activity with the lowest MIC and MBC values (Table 4). Chlorite anion exhibits potent antimicrobial activity, usually in the form of chlorine dioxide (ClO_2_) and is mainly used for disinfecting water and surfaces [18,19]. Although chlorine dioxide is generally considered safe, there are concerns about its toxicity for aquatic life [20], as well as rats [21] and humans [22].

A plausible explanation for the higher antibacterial activity of [Bmim][ClO_2_] is its lower ionicity, which encourages the formation of contact ion pairs or small aggregates with the imidazolium cation. These species are likely to partition more easily into lipid bilayers than fully dissociated ions, thereby raising the local concentration of the chlorite anion at the cell envelope. The combined effect of membrane-active [Bmim]^+^ and the redox reactivity of ClO_2_^−^ could lead to increased envelope damage at the interface, even though overall conductivity is lower. This mechanistic view aligns with reported membrane-targeting actions of imidazolium ILs and the known oxidative mode of action of chlorite/chlorine dioxide, although direct confirmation for [Bmim][ClO_2_] still needs to be provided [23,24,25,26].

### 2.4. ADMET Properties

**Figure 9:**
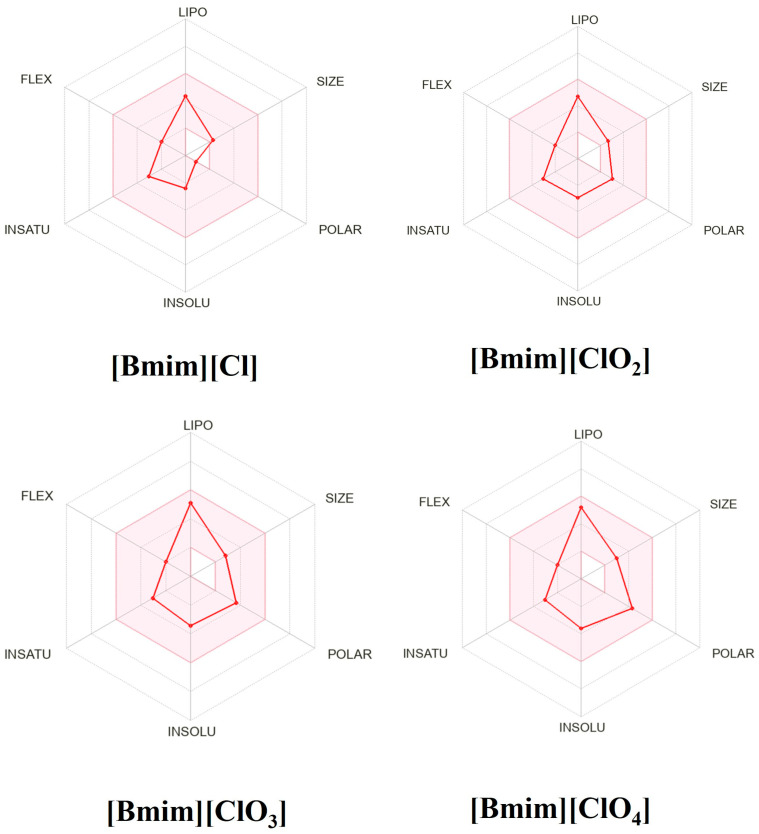
The bioavailability radars of drug-likeness properties of four ionic liquids. The pink area represents the optimal range for each property: XLOGP3 (lipophilicity); MW (molecular weight); TPSA (total polar surface area); INSOLU (log *S*, solubility in water); INSATU (saturation: fraction of carbons in the sp^3^ hybridization); FLEX (flexibility).

In silico pharmacokinetics, drug-likeness and medicinal-chemistry friendliness were computed for the four ionic liquids (Table 5). Drug-likeness was visualized with bioavailability radars (Figure 9). The optimal ranges used were lipophilicity XLOGP3 between −0.7 and 5.0; molecular weight 150–500 g·mol^−1^; polarity (TPSA) 20–130 Å^2^; aqueous solubility (log S) ≤ 6; saturation (fraction Csp^3^) ≥ 0.25; and flexibility (rotatable bonds) ≤ 9. All four compounds meet these physicochemical criteria and show 0 Lipinski violations [27].

Predicted absorption and distribution differ across the series. [Bmim][Cl] shows low human passive gastrointestinal absorption, whereas [Bmim][ClO_3_] and [Bmim][ClO_4_] are predicted to have low Caco-2 permeability despite high passive GI absorption. Predicted BBB permeability is absent for [Bmim][Cl] and [Bmim][ClO_4_], and present for [Bmim][ClO_2_] and [Bmim][ClO_3_] [28].

Safety-related flags require cautious interpretation. AMES mutagenicity is predicted to be positive for [Bmim][ClO_2_] and [Bmim][ClO_3_], while carcinogenicity is expected to be negative for all four. All compounds show high *Tetrahymena pyriformis* toxicity and are predicted to be biodegradable; fish, honey-bee, and rat acute toxicity are predicted to be low, with the same acute toxicity class (III). Taken together, the drug-likeness filters are satisfied, but the in silico mutagenicity flags for [Bmim][ClO_2_]/[Bmim][ClO_3_] and the GI/Caco-2/BBB patterns indicate that further toxicological and permeability studies are necessary before any pharmacological claims. In particular, although [Bmim][ClO_2_] shows strong antibacterial effects experimentally, its AMES-positive prediction precludes designating it as a promising drug candidate without additional optimization and validation.

Integrated experimental–computational results provide practical guidance for tailoring [Bmim]-based ionic liquids. Chlorate offers the highest conductivity at workable viscosity, perchlorate provides the greatest ionicity (Walden slope ≈ 1; α > 90%) although with higher viscosity, while chlorite shows lower ionicity and stronger ion pairing that can enhance interfacial antimicrobial action. Together, these trends define clear, application-oriented choices for electrolytes versus antimicrobial formulations and set the stage for targeted optimization in future work.

## 3. Materials and Methods

### 3.1. General Synthesis Procedure

**Figure 10:**
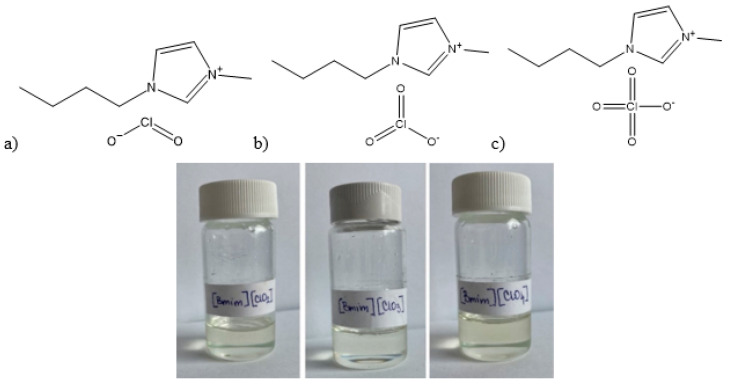
The structures and the appearance of the prepared ionic liquid: (**a**) [Bmim][ClO_2_] (**b**) [Bmim][ClO_3_] and (**c**) [Bmim][ClO_4_].

To synthesize 1-butyl-3-methylimidazolium chlorite [Bmim][ClO_2_], 1-butyl-3-methylimidazolium chlorate [Bmim][ClO_3_], and 1-butyl-3-methylimidazolium perchlorate [Bmim][ClO_4_], equimolar amounts of 1-butyl-3-methylimidazolium chloride [Bmim][Cl] (79917-90-1, TCI Europe N.V. (Tokyo Chemical Industry), Zwijndrecht, Belgium, ≥0.98), and the corresponding sodium or potassium oxychloride salts (sodium chlorite (7775-09-9, Sigma-Aldrich (Merck KGaA), Darmstadt, Germany, ≥0.99), potassium chlorate (3811-04-9, Sigma-Aldrich (Merck KGaA), Darmstadt, Germany, ≥0.99), or potassium perchlorate (7778-74-7, Sigma-Aldrich (Merck KGaA), Darmstadt, Germany, ≥0.99)) were measured and mixed in a one-neck flask with acetone (67-64-1, J.T. Baker B.V. (Avantor), Deventer, The Netherlands, ≥0.99) as a solvent. Acetone was chosen as ionic liquids dissolve in it, while the solubility of the product NaCl or KCl formed is very low, thus allowing for efficient precipitation. After two hours, the acetone solution of the ionic liquids was filtered through a G-4 filter, and excess acetone was removed by vacuum evaporation at elevated temperatures. Three light yellow ionic liquids were obtained (Figure 10). CAUTION! An attempted synthesis of 1-butyl-3-methylimidazolium hypochlorite, [Bmim][ClO], via synproportionation of [Bmim][Cl] and [Bmim][ClO_2_] was abandoned for safety reasons after the reaction mixture detonated upon mixing.

A silver nitrate spot test checked the presence of chloride. All three ionic liquids showed a negative chloride reaction and were then characterized by recording NMR and IR spectra, shown in Figures S1–S4 in the Supporting Information along with their adequate assignments. All three ionic liquids were isolated with >90% yield; the principal material losses occurred when the NaCl and KCl precipitate was removed in the metathesis step (filtration/washing). 1H NMR (D_2_O, 400 MHz) shows only the expected [Bmim]+ resonances with integral ratios matching the theoretical 1:2:3:2:2:2:3 pattern within ±5–7%, and no extraneous signals above baseline. On this basis, we confirm that the purity of each ionic liquid is not less than 95% by 1H NMR (estimate for the organic cation). Since chlorite, chlorate and perchlorate anions are oxidizing agents, Karl–Fisher titration could not be reliably performed for water determination. Instead, each ionic liquid was dried under high vacuum over P_2_O_5_ to constant mass, with successive drying cycles until the mass stabilized to ±0.0001 g (4 decimal spaces). Under these conditions, residual water was considered negligible for our measurements and great care was taken during storage and handling.

### 3.2. Apparatus and Their Procedures

#### 3.2.1. Nuclear Magnetic Resonance (NMR) and Infrared (IR) Spectra

The NMR spectra of ionic liquids were obtained at a temperature of *T* = 298.15 K in D_2_O using a Bruker Advance III 400 MHz spectrometer (Bruker BioSpin GmbH, Rheinstetten, Germany). For calibrating chemical shifts for ^1^H and ^13^C, tetramethylsilane was used as an internal standard. The obtained NMR spectra were assigned using 1H homodecoupling and the 2D COSY method. For assigning ^13^C NMR spectra, the selective decoupling technique was used. Infrared spectra were recorded on a Thermo Nicolet Nexus 670 spectrometer (Thermo Nicolet Corporation (Thermo Fisher Scientific), Madison, WI, USA) fitted with a Universal ATR Sampling Accessory. The range of wavenumber was from 4000 to 650 cm^−1^, and the measurements were performed with 60 scans at room temperature, with a spectral resolution of 2 cm^−1^. The droplet of ionic liquid was placed on top of a germanium crystal. The software package Omnic version 6.2 was used for data acquisition and spectral analysis.

#### 3.2.2. Density, Viscosity, and Electrical Conductivity Measurements

The density measurements were carried out using the Rudolph Research Analytical vibrating tube densimeter (Rudolph Research Analytical, Hackettstown, NJ, USA). Thermal control was achieved through inbuilt Peltier regulation with a precision of 0.05 K. Calibration of the densimeter was performed before each series of measurements, and ambient air and bi-distilled ultra-pure water were used at atmospheric pressure (*p* = 0.1 MPa) and in the temperature range of *T* = 293.15 K to 323.15 K. Density values used were the mean of three measurements with reproducibility of 0.001% and standard uncertainty of the measurements was less than 3.0 × 10^−3^ g·cm^−3^.

Viscosity measurements of the studied ionic liquids were performed by a Brookfield Viscometer DV II + Pro (AMETEK Brookfield (Brookfield Engineering Laboratories), Middleborough, MA, USA) with thermal control provided by a connected Lauda E 100 thermostat (LAUDA DR. R. WOBSER GmbH & Co. KG, Lauda-Königshofen, Germany) with ±0.01 K precision. The measurement cell was filled with about 8 cm^3^ of pure ionic liquid and the spindle (type SC4-18) was immersed within. Rate of rotation of the spindle was set to obtain adequate torque. The viscometer cell was protected from moisture with a cap that allowed for unobstructed spindle rotation. The instrument was calibrated using a set of liquids with different viscosities, and these standards were purchased from the instrument’s manufacturer. Experimental values presented represent the mean of several measurements.

The electrical conductivity measurements for pure ILs were conducted in a Pyrex cell with platinum electrodes and a Jenco 3107 conductivity meter (Jenco Instruments, Inc., San Diego, CA, USA). The cell constant was determined to be 1.0353 cm^–1^. The relative standard uncertainty for electrical conductivity was less than 1.5%, and all experimental values represent the mean of three measurements. The results were analyzed using Origin 8.5.1 software.

#### 3.2.3. Computational Details

The Jaguar 9.0 program of the Schrödinger Materials Science Suite 2015-4 package was used to perform the DFT calculations. Temperature of the simulations was set as 298.15 K. Hessian analysis was used to check the optimized structures and only results with no imaginary wavelengths were considered for further DFT analysis. The dispersion-corrected B3LYP-d3 functional was applied with basis set 6-31G+ (d,p), along with superposition error (BSSE) which included the Boys–Bernardi counterpoise method [29]. The detection and analysis of non-covalent interactions (NCIs) from optimized structures were performed using the Johnson et al. [30] method.

#### 3.2.4. Calculation of ADMET Properties

Absorption, distribution, metabolism, excretion, and toxicity (ADMET) properties were calculated using a web tool SwissADME freely available at http://www.swissadme.ch (accessed on 2 July 2025) [31] and ADMETlab 2.0 (https://admetmesh.scbdd.com/service/screening/index) (accessed on 2 July 2025) [32]. SMILES notations were used as an input for molecular structures.

#### 3.2.5. Antimicrobial Testing Methods

The antimicrobial activity of investigated ionic liquids (IL) was tested against three bacterial (*Escherichia coli* ATCC 8739, *Staphylococcus aureus* ATCC 33592, *Bacillus cereus* ATCC11778) and one yeast species (*Candida quilliermondii* JR-23). All tests were assessed in triplicate using the in vitro broth microdilution method in a 96-well microtiter plate. The methodology followed the adapted CLSI/EUCAST guidelines [33,34,35].

C. *quilliermondii* was cultivated on malt extract agar (Torlak, Serbia) for 48 h at 37 °C, while bacterial species were pre-cultured on Mueller–Hinton agar (Institute of Virology, Vaccines and Sera “Torlak”—Belgrade, Serbia) for 24 h at 37 °C. For bacterial and yeast cultures, saline suspensions with a turbidity equivalent to a 0.5 McFarland standard were prepared. The experiment was conducted in sterile polypropylene 96-well microtiter plates with rounded bottoms (Spektar, Čačak, Serbia). In each well, 50 µL of MB (malt broth, Institute of Virology, Vaccines and Sera “Torlak”—Belgrade, Serbia), 1 µL of saline suspension, and 50 µL of ILs were added. The concentrations of tested ILs ranged from 0.78 mg/mL to 107.1 mg/mL. The growth in each test-well was compared to that of the growth control (IL-free) well after an incubation period (24 h at 37 °C for bacteria and 48 h at 37 °C for yeasts) and MIC values were determined for all tested microorganisms.

The content of test wells where no visible growth was detected was transferred to Petri plates with Mueller–Hinton agar/Malt extract agar and incubated for 24 h (for bacteria) or 48 h (for yeasts) at 37 °C to evaluate minimal bactericidal and minimal fungicidal concentrations of ILs (MBC and MFC). The lowest IL concentration, MBC, and MFC values were found to have a 99.9% reduction in the viability of the original fungal inoculum. A commercial ionic liquid, 1-butyl-3-methylimidazolium chloride ([Bmim]Cl), was included for benchmarking as it serves as a positive control for the cationic baseline and a comparator for anion effects. It possesses an intrinsic antimicrobial activity due to membrane perturbation by the BMIM cation, and chloride is non-oxidizing. This comparator enables direct assessment of how anion oxygenation modulates antimicrobial potency relative to the chloride analogue.

## 4. Conclusions

Three ionic liquids, 1-butyl-3-methylimidazolium chlorite, chlorate, and perchlorate, were synthesized and characterized. The density decreases linearly with temperature for all three; at any given temperature, [Bmim][ClO_4_] is the most dense, and [Bmim][ClO_2_] the least. Viscosity decreases as temperature rises, and the thermal expansion coefficients increase with temperature, with the order α_p_([Bmim][ClO_4_]) < α_p_([Bmim][ClO_2_]) < α_p_([Bmim][ClO_3_]). These trends suggest lower expansivity for the perchlorate salt and are consistent with smaller molar volumes for [Bmim][ClO_4_] across the studied temperature range.

Electrical and molar conductivities are highest for the chlorate salt, reflecting an optimal balance between ionicity and viscous drag across the series. Arrhenius analysis shows that the viscous-flow activation energy is most significant for [Bmim][ClO_2_], indicating the strongest temperature dependence of viscosity. Conductivity activation energies are highest for [Bmim][ClO_4_] and are very similar for [Bmim][ClO_2_] and [Bmim][ClO_3_].

Walden analysis shows that [Bmim][ClO_3_] and [Bmim][ClO_4_] are close to the ideal KCl line with ionicity above 90 percent, while [Bmim][ClO_2_] has significantly lower ionicity that decreases with temperature. Therefore, for the chlorate and perchlorate ionic liquids, molar conductivity is mainly influenced by viscosity, whereas for the chlorite ionic liquid, it depends on viscosity and the extent of dissociation.

Computational results support these observations. Optimized structures and molecular electrostatic potential maps reveal directional C–H···O contacts and more localized negative potential on chlorite oxygen atoms, consistent with stronger specific cation–anion interactions. Frontier-orbital analysis shows the largest HOMO–LUMO gap for [Bmim][ClO_4_] and the smallest for [Bmim][ClO_2_], indicating the hardest electronic profile for perchlorate and the softest for chlorite. Thermochemical descriptors suggest stronger specific stabilization in the chlorite ion pair and the weakest in the perchlorate pair.

In antimicrobial tests, [Bmim][ClO_2_] demonstrated the strongest activity against the tested strains. A likely explanation is its lower ionicity, which promotes contact ion pairs or small aggregates that more easily penetrate cell envelopes, improving local delivery of the chlorite anion at the interface. All four ionic liquids passed in silico ADMET screening (Lipinski and bioavailability radar criteria); however, AMES mutagenicity was predicted for [Bmim][ClO_2_] and [Bmim][ClO_3_]. Therefore, although [Bmim][ClO_2_] has the highest antibacterial effect, further toxicological testing and optimization are necessary before considering it as a drug candidate.

## Figures and Tables

**Table 1:** Experimental values of density (*d*), viscosity (*η*), and electrical conductivity (*κ*), and calculated values of thermal expansion coefficients (α_p_), molar conductivity (*Λ*), and ionicity (α) in the temperature range from *T* = 293.15 to 323.15 K.

Property	Ionic Liquid	293.15	298.15	303.15	308.15	313.15	318.15	323.15
***d*** **(g·cm^−3^)**	[Bmim][ClO_2_]	1.11476	1.11182	1.10879	1.10572	1.10250	1.09914	1.09576
	[Bmim][ClO_3_]	1.19526	1.19184	1.18852	1.18509	1.18173	1.17813	1.17448
	[Bmim][ClO_4_]	1.25649	1.25267	1.24921	1.24586	1.24205	1.23876	1.23543
***α*_p_∙10^4^** **(K^–1^)**	[Bmim][ClO_2_]	5.697	5.712	5.728	5.744	5.761	5.778	5.796
	[Bmim][ClO_3_]	5.776	5.793	5.809	5.826	5.843	5.861	5.879
	[Bmim][ClO_4_]	5.580	5.597	5.613	5.628	5.645	5.660	5.675
***η*** **(mPa·s)**	[Bmim][ClO_2_]	42.57	26.71	15.24	6.79	3.69	2.14	1.24
	[Bmim][ClO_3_]	109.95	86.58	67.09	54.21	44.05	36.13	30.49
	[Bmim][ClO_4_]	227.96	188.12	136.47	104.12	78.11	64.87	55.85
***κ*** **(mS·cm^−1^)**	[Bmim][ClO_2_]	1.87	2.33	2.94	3.68	4.53	5.46	6.50
	[Bmim][ClO_3_]	2.33	3.04	3.86	4.80	5.82	7.10	8.41
	[Bmim][ClO_4_]	1.27	1.73	2.30	2.97	3.76	4.66	5.63
***Λ*** **(S·** **cm^2^·** **mol^−1^)**	[Bmim][ClO_2_]	0.347	0.433	0.548	0.688	0.849	1.027	1.226
	[Bmim][ClO_3_]	0.434	0.568	0.723	0.902	1.097	1.342	1.594
	[Bmim][ClO_4_]	0.241	0.330	0.440	0.569	0.723	0.898	1.088
***α*** **(%)**	[Bmim][ClO_2_]	67.24	65.85	62.27	57.51	51.20	43.18	35.21
	[Bmim][ClO_3_]	92.56	93.21	92.76	92.98	93.18	93.32	93.05
	[Bmim][ClO_4_]	94.76	96.05	95.98	95.86	95.69	95.64	95.71

**Table 2:** Calculated values of density fitting coefficients (*a* and *b*) with regression coefficient (*R*^2^) from Equation (1), activation energy of the viscous flow (*E*_a_)*,* and conductivity activation energy (*E*’_a_).

Parameter	[Bmim][ClO_2_]	[Bmim][ClO_3_]	[Bmim][ClO_4_]
***a* (g·cm^−3^)**	1.3012	1.3977	1.4618
***b*∙10^4^ (g·cm^−3^·K^−1^)**	−6.3514	−6.9043	−7.0114
***R* ^2^**	0.9994	0.9997	0.9995
***E_a_* (kJ·mol^−1^)**	96.03	33.89	38.92
***E*’*_a_* (kJ·mol^−1^)**	33.55	34.06	39.59

**Table 3:** Thermodynamic parameters of ion pairing with BSSE correction for the investigated ionic liquids.

Parameter	[Bmim][ClO_2_]	[Bmim][ClO_3_]	[Bmim][ClO_4_]
**Δ*G* (kJ·mol^−1^)**	−26.762	−28.366	−29.484
**Δ*H* (kJ·mol^−1^)**	9.625	10.41	10.932
**Δ*S* (J·mol^−1^·K^−1^)**	122.045	130.057	135.554

**Table 4:** MIC and MBC/MFC values of tested ILs.

Tested Microorganisms	[Bmim][Cl]		[Bmim][ClO_2_]		[Bmim][ClO_3_]		[Bmim][ClO_4_]	
	MIC	MBC /MFC	MIC	MBC /MFC	MIC	MBC /MFC	MIC	MBC /MFC
	mg·mL^−1^		mg·mL^−1^		mg·mL^−1^		mg·mL^−1^	
**Bacteria**								
*Escherichia coli*	5.90	/	24.97	99.90	26.77	107.1	28.12	1112.5
*Staphylococcus aureus*	/	/	24.97	49.95	53.55	107.1	28.12	56.25
*Bacillus cereus*	/	/	99.90	99.90	107.1	107.1	112.5	112.5
**Yeasts**								
*Candida guillermondii*	/	/	49.95	49.95	107.1	107.1	56.25	56.25

**Table 5:** Absorption, distribution, metabolism, excretion, and toxicity (ADMET) properties of studied Ils.

ADMET Property	[Bmim][Cl]	[Bmim][ClO_2_]	[Bmim][ClO_3_]	[Bmim][ClO_4_]
MW (g/mol)	174.67	206.67	222.67	238.67
XLOGP3	2.13	2.76	3.41	3.58
TPSA (Å^2^)	8.81	48.94	66.01	83.08
Fraction Csp^3^	0.62	0.62	0.62	0.62
RB	3	3	3	3
logS	−2.4	−2.95	−3.44	−3.62
Wat. sol.	Soluble	Soluble	Soluble	Soluble
HGI absorp.	Low	High	High	High
BBB perm.	No	Yes	Yes	No
Caco-2 perm.	Yes	Yes	No	NO
Druglikeness	Yes; 0 violation	Yes; 0 violation	Yes; 0 violation	Yes; 0 violation
AMES tox.	No	Yes	Yes	No
Carcinogens	No	No	No	No
Fish tox.	Low	Low	Low	Low
*Tetrahymena pyriformis* tox.	High	High	High	High
Honey bee tox.	Low	Low	Low	Low
Biodegradation	Yes	Yes	Yes	Yes
Acute oral tox.	III. class	III. class	III. class	III. class
Rat acute toxicity, (LD_50_, mol/kg)	2.94	2.88	2.81	2.84

MW (molecular weight); XLOGP3 (lipophilicity descriptor); TPSA (topological polar surface area); Fract. Csp3 (proportion of sp^3^-hybridized carbon atoms to the total number of carbon atom); RB (number of rotatable bonds); logS (logarithm of the molar solubility in water); Wat. sol. (solubility in water); HGI absorb. (human passive gastrointestinal absorption; BBB perm. (blood–brain barrier permeability); Caco-2 perm. (human colon adenocarcinoma cell line) permeability; Drug-likeness (Lipinski role); AMES tox. (Ames test for mutagenicity).

## Data Availability

Data will be made available from the authors upon request.

## Supplementary Materials

The following supporting information can be downloaded at: https://www.mdpi.com/article/10.3390/molecules30224346/s1, Figure S1: IR Spectra; Figure S2: (**a**) 1H spectrum gained by 1H homodecoupling and the 2D COSY method and (**b**) 13C NMR spectrum gained by selective decoupling technique for [Bmim][ClO_2_]; Figure S3: (**a**) 1H spectrum gained by 1H homodecoupling and the 2D COSY method and (**b**) 13C NMR spectrum gained by selective decoupling technique for [Bmim][ClO_3_]; Figure S4: (**a**) 1H spectrum gained by 1H homodecoupling and the 2D COSY method and (**b**) 13C NMR spectrum gained by selective decoupling technique for [Bmim][ClO_4_].
