# Supplementary material for: Synthesis, Physicochemical Characterization, Antimicrobial Properties, and DFT/ADMET Calculations of Imidazolium-Based Ionic Liquids with a Homologous Series of Oxychlorine Anions
Source: Molecules. 2025 Nov 10;30(22):4346. doi: 10.3390/molecules30224346 (PMC12654788; doi:10.3390/molecules30224346)
Supplement: Supplementary file 1 [file molecules-30-04346-s001.zip › molecules-3961296-supplementary.pdf]

SUPPORTING INFORMATION

Synthesis, Physicochemical Characterization, Antimicrobial Properties, and DFT/ADMET Calculations of Imidazolium-Based Ionic Liquids with a Homologous Series of Oxychlorine Anions

Milan B. Vraneš[1], Eleonora Čapelja[2], Maja Karaman[2], Teona Teodora Borović[1], Andrija Vukov[1], Sara Klimenta[1], Vesna Rastija[3], Jovana J. Selak[1]

[1] *Department of Chemistry, Biochemistry and Environmental Protection, Faculty of Science, University of Novi Sad, Trg Dositeja Obradovića 3, 21000 Novi Sad, Serbia*

[2] *University of Novi Sad, Faculty of Sciences, Department of Biology and Ecology, Trg Dositeja Obradovića 2, Novi Sad, 21000, Serbia*

[3] *Department of Agroecology and Environmental Protection, Faculty of Agrobiotechnical Sciences Osijek, Josip Juraj Strossmayer University of Osijek, Vladimira Preloga 1, HR-31000 Osijek, Croatia*

IR and NMR assignment for the three Ionic Liquids are as follows:

[Bmim][ClO$_2$] – IR: 3029-2986 (symmetric stretching of C-H in N-CH_3); 2948 (symmetric stretching of C-H from -CH_3- and -CH_2-); 1355 (rocking of H–C–H in butyl group); 1178 (sceletal vibrations of imidazolium ring); 955 (symmetric stretching of O-Cl); 731 (wagging of CH=CH of imidazolium ring). ^{1}H NMR (D_2O): 0.79 (t, 3H, *J*= 7.1 Hz, $NCH_2CH_2CH_2C\boldsymbol{H}_3$); 1.16 (m, 2H, $NCH_2CH_2C\boldsymbol{H}_2CH_3$); 1.71 (m, 2H, $NCH_2C\boldsymbol{H}_2CH_2CH_3$);3.69 (s, 3H, NCH_3); 3.96 (t, 2H, $NC\boldsymbol{H}_2CH_2CH_2CH_3$); 7.11-7.25 (m, 3H, H-3, H-5, H-2); 8.44 (s, 1H, H-2). ^{13}C NMR: 16.05 ($NCH_2CH_2CH_2\boldsymbol{C}H_3$); 20.31 ($NCH_2CH_2\boldsymbol{C}H_2CH_3$); 32.94 ($NCH_2\boldsymbol{C}H_2CH_2CH_3$); 39.15 ($N\boldsymbol{C}H_3$); 50.86 ($N\boldsymbol{C}H_2CH_2CH_2CH_3$).

[Bmim][ClO$_3$] – IR: 3031-2982 (symmetric stretching of C-H in N-CH_3); 2942 (symmetric stretching of C-H from -CH_3- and -CH_2-); 1351 (rocking of H–C–H in butyl group); 1172 (sceletal vibrations of imidazolium ring); 930 (symmetric stretching of O-Cl); 731 (wagging of CH=CH of imidazolium ring). ^{1}H NMR (D_2O): 0.80 (t, 3H, *J*= 7.1 Hz, $NCH_2CH_2CH_2C\boldsymbol{H}_3$); 1.21 (m, 2H, $NCH_2CH_2C\boldsymbol{H}_2CH_3$); 1.75 (m, 2H, $NCH_2C\boldsymbol{H}_2CH_2CH_3$);3.77 (s, 3H, NCH_3); 4.09 (t, 2H, $NC\boldsymbol{H}_2CH_2CH_2CH_3$); 7.16-7.34 (m, 3H, H-3, H-5, H-2); 8.64 (s, 1H, H-2). ^{13}C NMR: 16.26 ($NCH_2CH_2CH_2\boldsymbol{C}H_3$); 20.93 ($NCH_2CH_2\boldsymbol{C}H_2CH_3$); 33.15 ($NCH_2\boldsymbol{C}H_2CH_2CH_3$); 39.41 ($N\boldsymbol{C}H_3$); 51.08 ($N\boldsymbol{C}H_2CH_2CH_2CH_3$).

[Bmim][ClO$_4$] – IR: 3029-2981 (symmetric stretching of C-H in N-CH_3); 2939 (symmetric stretching of C-H from -CH_3- and -CH_2-); 1351 (rocking of H–C–H in butyl group); 1173 (sceletal vibrations of imidazolium ring); 1084 (symmetric stretching of O=Cl); 731 (wagging of CH=CH of imidazolium ring). ^{1}H NMR (D_2O): 0.85 (t, 3H, *J*= 7.1 Hz, $NCH_2CH_2CH_2C\boldsymbol{H}_3$); 1.24 (m, 2H, $NCH_2CH_2C\boldsymbol{H}_2CH_3$); 1.75 (m, 2H, $NCH_2C\boldsymbol{H}_2CH_2CH_3$);3.75 (s, 3H, NCH_3); 4.05 (t, 2H, $NC\boldsymbol{H}_2CH_2CH_2CH_3$); 7.16-7.37 (m, 3H, H-3, H-5, H-2); 8.52 (s, 1H, H-2). ^{13}C NMR: 16.31 ($NCH_2CH_2CH_2\boldsymbol{C}H_3$); 20.55 ($NCH_2CH_2\boldsymbol{C}H_2CH_3$); 33.18 ($NCH_2\boldsymbol{C}H_2CH_2CH_3$); 39.41 ($N\boldsymbol{C}H_3$); 51.08 ($N\boldsymbol{C}H_2CH_2CH_2CH_3$).

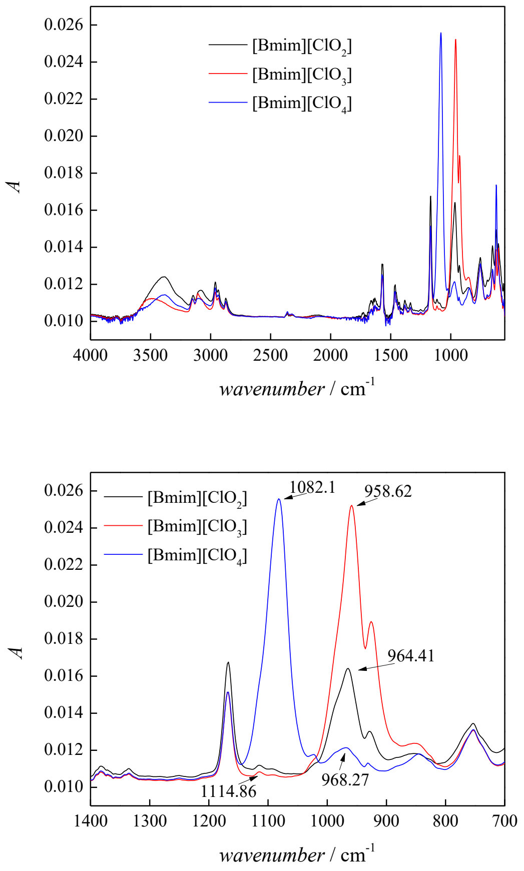

Figure S1. IR spectra.

a)

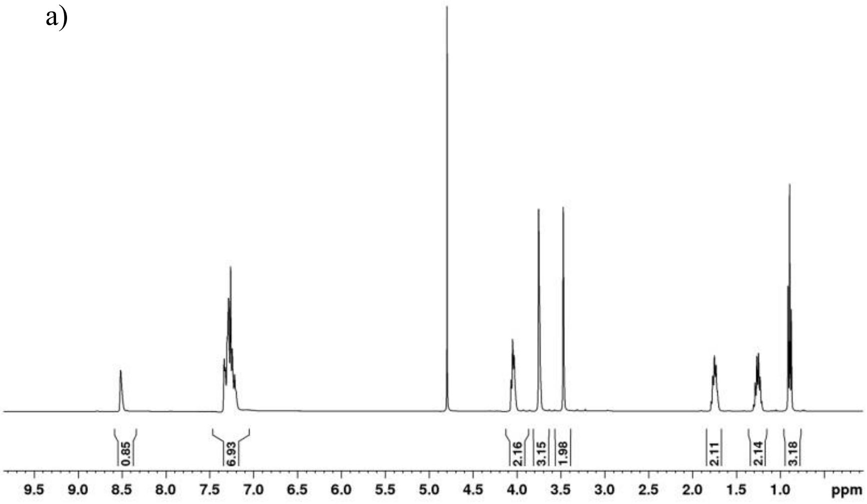

b)

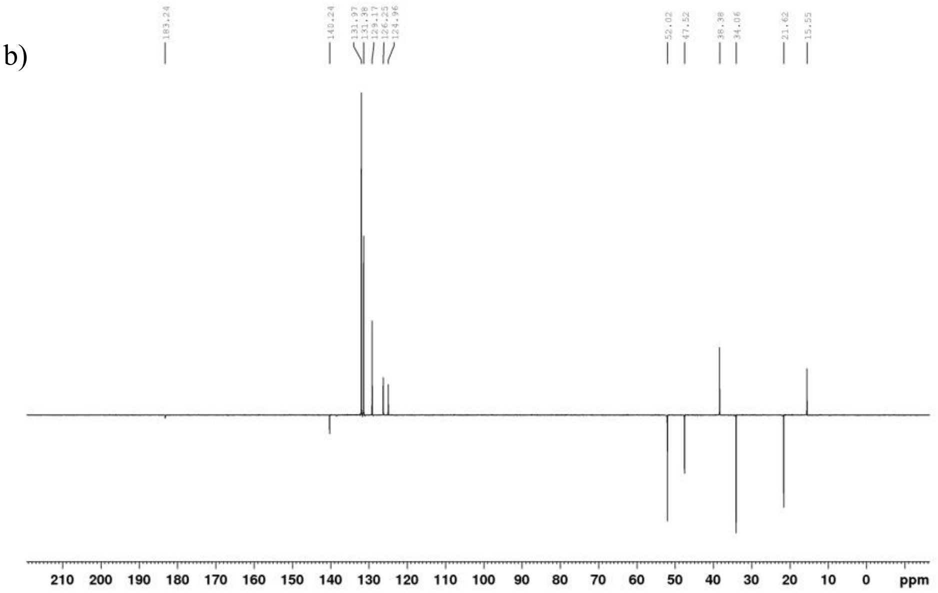

Figure S2. a) ^{1}H spectrum gained by 1H homodecoupling and the 2D COSY method and b) ^{13}C NMR spectrum gained by selective decoupling technique for [Bmim][ClO_2].

a)

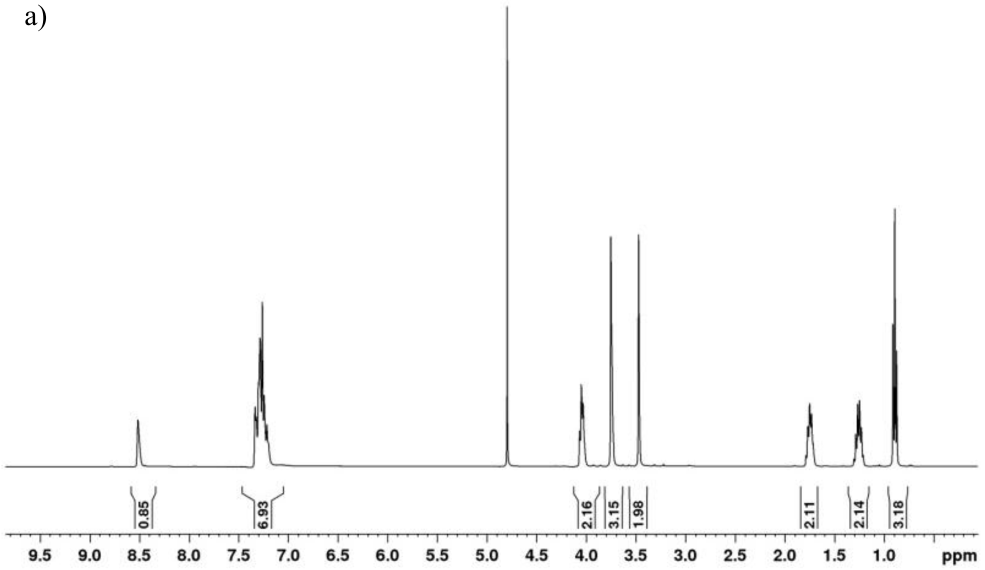

b)

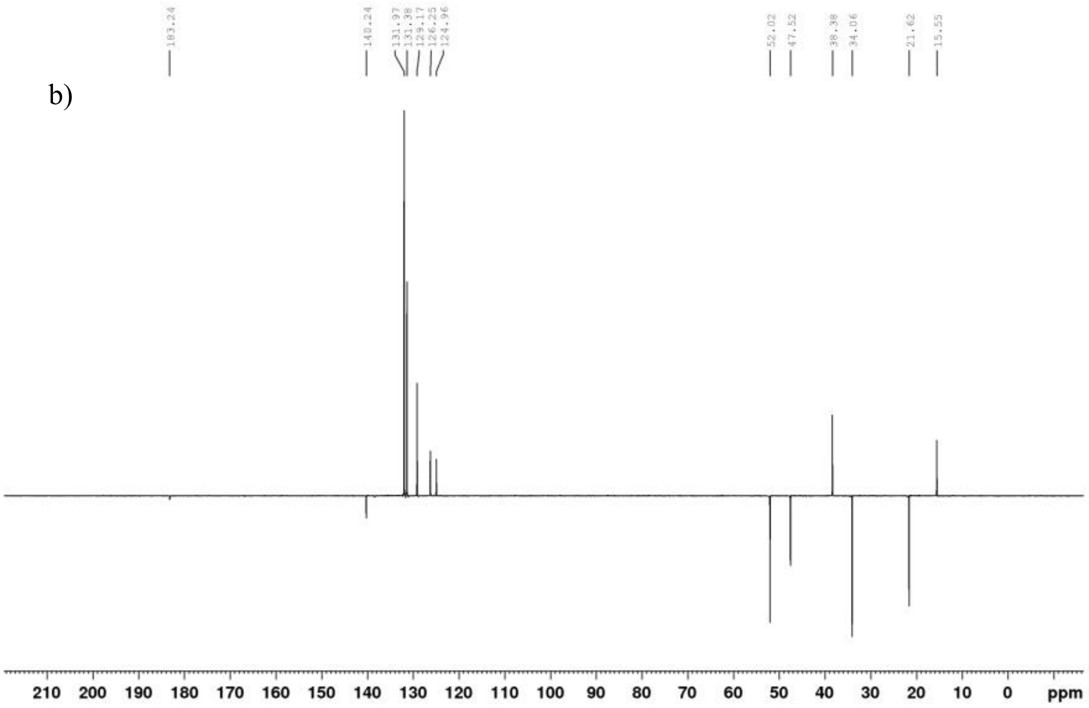

Figure S3. a) ^{1}H spectrum gained by 1H homodecoupling and the 2D COSY method and b) ^{13}C NMR spectrum gained by selective decoupling technique for [Bmim][ClO_3].

a)

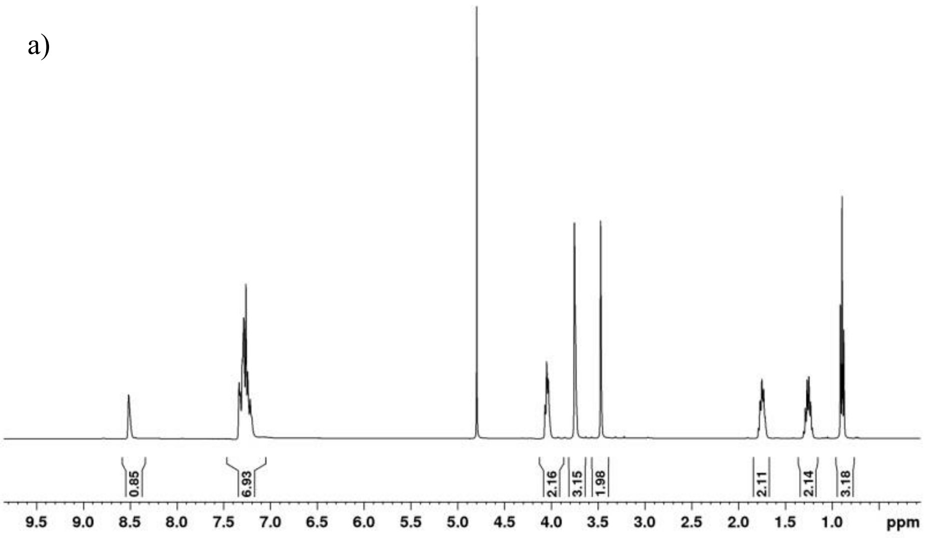

b)

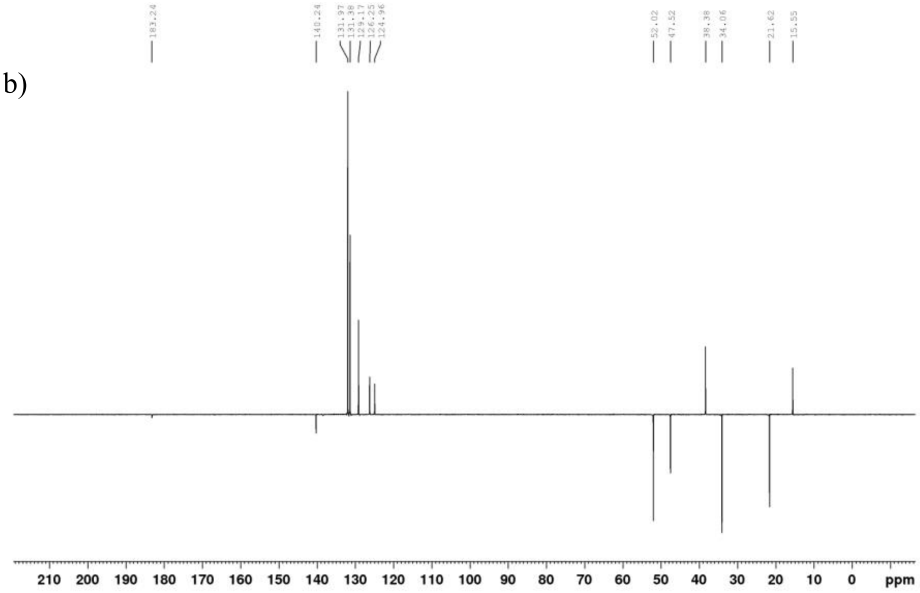

Figure S4. a) ^{1}H spectrum gained by 1H homodecoupling and the 2D COSY method and b) ^{13}C NMR spectrum gained by selective decoupling technique for [Bmim][ClO_4].
